# Supplementary material for: Temporal Change in Biomarkers of Bone Turnover Following Late Evening Ingestion of a Calcium-Fortified, Milk-Based Protein Matrix in Postmenopausal Women with Osteopenia
Source: Nutrients. 2019 Jun 23;11(6):1413. doi: 10.3390/nu11061413 (PMC6627915; doi:10.3390/nu11061413)
Supplement: Supplementary file 1 [file nutrients-11-01413-s001.zip › CONSORT FLOW CHART.pptx]

## Slide 1
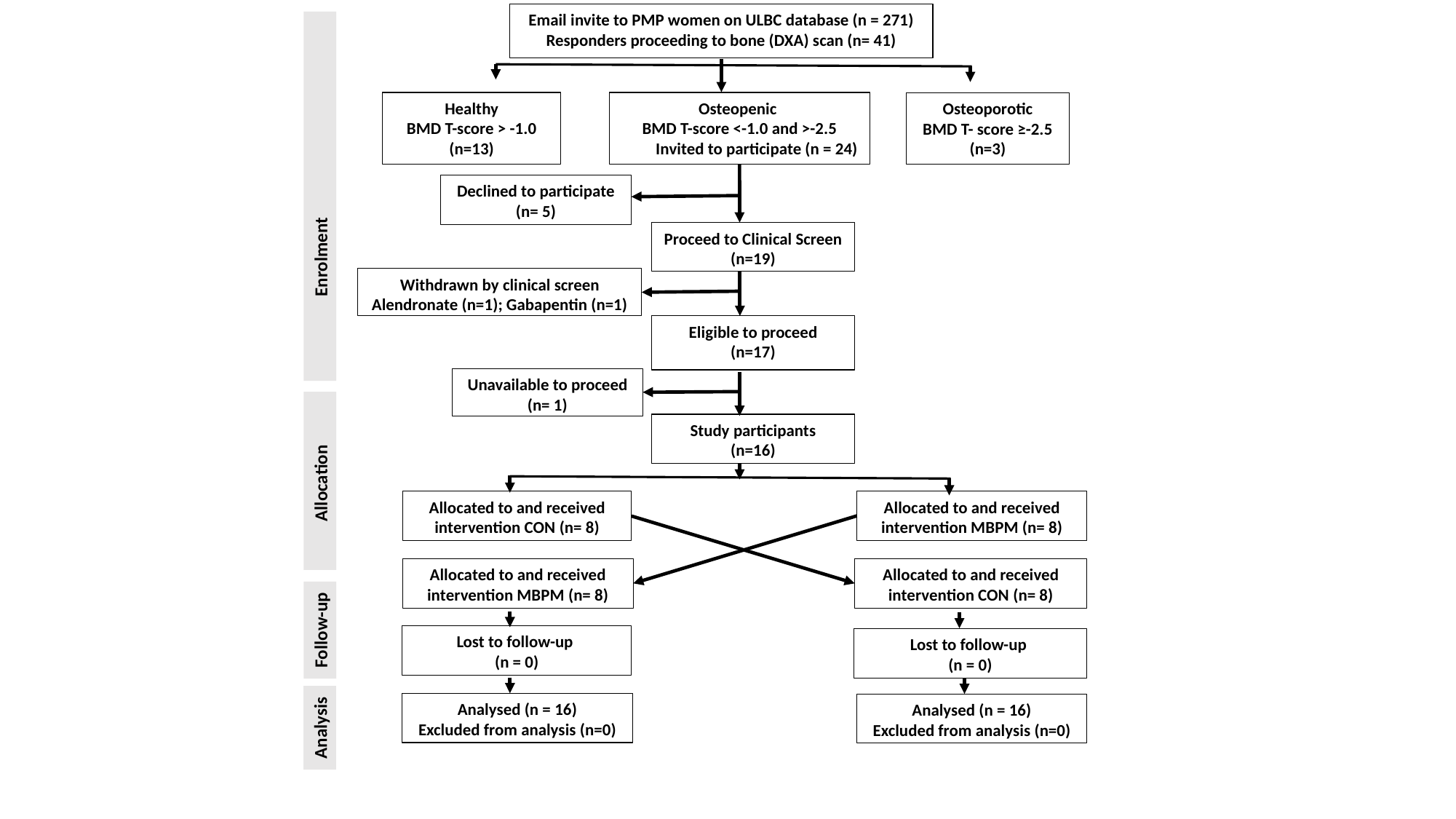

Email invite to PMP women on ULBC database (n = 271)
Responders proceeding to bone (DXA) scan (n= 41)
Healthy
BMD T-score > -1.0
(n=13)
Osteopenic
BMD T-score <-1.0 and >-2.5
 Invited to participate (n = 24)
Osteoporotic
BMD T- score ≥-2.5
(n=3)
Declined to participate
(n= 5)
 Enrolment
Proceed to Clinical Screen
(n=19)
Withdrawn by clinical screen
Alendronate (n=1); Gabapentin (n=1)
Eligible to proceed
(n=17)
Unavailable to proceed
(n= 1)
Study participants
(n=16)
 Allocation
Allocated to and received intervention MBPM (n= 8)
Allocated to and received intervention CON (n= 8)
Allocated to and received intervention MBPM (n= 8)
Allocated to and received intervention CON (n= 8)
Follow-up
Lost to follow-up
(n = 0)
Lost to follow-up
(n = 0)
Analysed (n = 16)
Excluded from analysis (n=0)
Analysed (n = 16)
Excluded from analysis (n=0)
Analysis
