# Supplementary material for: Temporal Change in Biomarkers of Bone Turnover Following Late Evening Ingestion of a Calcium-Fortified, Milk-Based Protein Matrix in Postmenopausal Women with Osteopenia
Source: Nutrients. 2019 Jun 23;11(6):1413. doi: 10.3390/nu11061413 (PMC6627915; doi:10.3390/nu11061413)
Supplement: Supplementary file 1 [file nutrients-11-01413-s001.zip › Supplementary Table 1.docx]

**Supplementary Table S1:** Mass and nutrient composition of the proprietary products MBPM and CON.


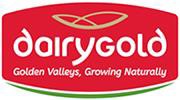


**Vanilla Flavoured Maltodextrin (CON)**

Product Description: Dry Maltodextrin 01955 with vanilla flavour

Directions for Use: Mix 52g powder sachet with 220ml water at 50°C and stir for 60 seconds. Consume either warm or cold.

Ingredient Declaration: Maltodextrin, vanilla flavouring

Physical & Organoleptic: Appearance: Free flowing powder

Colour: Uniform white colour throughout

Odour/taste: Sweet vanilla odour, slight sweet vanilla flavour

Country of Origin: Ireland


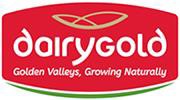


**High Protein Bone Health Powder Instant and Fortified (MBPM)**

Product Name: High Protein Bone Health Powder, Instant and Fortified

Product Description: Ready to mix protein based blend for Bone Health, instant and fortified with calcium and vitamin D.

Directions for Use: Mix 55g powder sachet with 220ml water at 50°C and stir for 60 seconds.

Ingredient Declaration: Milk & Whey protein, sugar, calcium, flavouring, vitamin D

Physical & Organoleptic: Appearance: Free flowing powder, free from lumps

Colour: Uniform colour throughout, dependant on flavour

Odour/taste: Free from foreign odours, dependant on flavour

Country of Origin: Ireland

Product Analysis (per 100g powder)

| Parameter | unit | MBPM | CON |
| --- | --- | --- | --- |
| Energy | kcal | 350 | 380 |
| Protein | % | 46.6 | N/A |
| Carbohydrate | % | 40.6 | 95.0 |
| Fat | % | 0.07 | N/A |
| Moisture (max) | % | 4.0 | 4.0 |
| Ash | % | 8.1 | N/A |
| Vitamin D3 | IU/100g | 80 | N/A |
| Calcium | mg/100g | 1840 | N/A |
