# Supplementary material for: Temporal Change in Biomarkers of Bone Turnover Following Late Evening Ingestion of a Calcium-Fortified, Milk-Based Protein Matrix in Postmenopausal Women with Osteopenia
Source: Nutrients. 2019 Jun 23;11(6):1413. doi: 10.3390/nu11061413 (PMC6627915; doi:10.3390/nu11061413)
Supplement: Supplementary file 1 [file nutrients-11-01413-s001.zip › Supplementary Table 2.docx]

**Supplementary Table S2**: Mass and nutrient composition of MBPM relative to participants’ body mass providing 0.33g protein per kilogram of body mass.

|  | **High Protein Bone Health Powder Instant and Fortified (MBPM)** | | | | | | | |
| --- | --- | --- | --- | --- | --- | --- | --- | --- |
|  | BM (kg) | MPBS mass (g) | Energy (kcal) | Protein (g) | Carbohydrate (g) | Fat (g) | Calcium (mg) | Vitamin D (ug) |
|  | 69.4 | 49.1 | 172 | 22.9 | 20.0 | 0.03 | 904 | 0.98 |
|  | 59.5 | 42.1 | 147 | 19.6 | 17.1 | 0.03 | 775 | 0.84 |
|  | 83 | 58.8 | 206 | 27.4 | 23.9 | 0.04 | 1081 | 1.18 |
|  | 70.2 | 49.7 | 174 | 23.2 | 20.2 | 0.03 | 915 | 0.99 |
|  | 73.5 | 52.0 | 182 | 24.3 | 21.1 | 0.04 | 958 | 1.04 |
|  | 54 | 38.2 | 134 | 17.8 | 15.5 | 0.03 | 704 | 0.76 |
|  | 91.1 | 64.5 | 226 | 30.1 | 26.2 | 0.05 | 1187 | 1.29 |
|  | 75.7 | 53.6 | 188 | 25.0 | 21.8 | 0.04 | 986 | 1.07 |
|  | 72.6 | 51.4 | 180 | 24.0 | 20.9 | 0.04 | 946 | 1.03 |
|  | 63.5 | 45.0 | 157 | 21.0 | 18.3 | 0.03 | 827 | 0.90 |
|  | 60.9 | 43.1 | 151 | 20.1 | 17.5 | 0.03 | 794 | 0.86 |
|  | 58 | 41.1 | 144 | 19.1 | 16.7 | 0.03 | 756 | 0.82 |
|  | 48 | 34.0 | 119 | 15.8 | 13.8 | 0.02 | 625 | 0.68 |
|  | 66.6 | 47.2 | 165 | 22.0 | 19.1 | 0.03 | 868 | 0.94 |
|  | 64.8 | 45.9 | 161 | 21.4 | 18.6 | 0.03 | 844 | 0.92 |
|  | 63.8 | 45.2 | 158 | 21.1 | 18.3 | 0.03 | 831 | 0.90 |
| Mean | 67.2 | 47.6 | 166 | 22.2 | 19.3 | 0.03 | 875 | 0.95 |
| SD | 10.4 | 7.4 | 26 | 3.4 | 3.0 | 0.01 | 136 | 0.15 |
| Min | 48.0 | 34.0 | 119 | 15.8 | 13.8 | 0.02 | 625 | 0.68 |
| Max | 91.1 | 64.5 | 226 | 30.1 | 26.2 | 0.05 | 1187 | 1.29 |
