# Supplementary material for: Temporal Change in Biomarkers of Bone Turnover Following Late Evening Ingestion of a Calcium-Fortified, Milk-Based Protein Matrix in Postmenopausal Women with Osteopenia
Source: Nutrients. 2019 Jun 23;11(6):1413. doi: 10.3390/nu11061413 (PMC6627915; doi:10.3390/nu11061413)
Supplement: Supplementary file 1 [file nutrients-11-01413-s001.zip › Supplementary Table 3.docx]

**Supplementary Table S3**: Mass and nutrient composition of CON matched for nutrient energy provided by MBPM

|  | **Vanilla Flavoured Maltodextrin (CONTROL)** | | | | | | | |
| --- | --- | --- | --- | --- | --- | --- | --- | --- |
|  | BM (kg) | CON mass (g) | Energy (kcal) | Protein (g) | Carbohydrate (g) | Fat (g) | Calcium (mg) | Vitamin D (ug) |
|  | 69.4 | 45.3 | 172 | 0 | 43.0 | 0 | 0 | 0 |
|  | 59.5 | 38.8 | 147 | 0 | 36.9 | 0 | 0 | 0 |
|  | 83 | 54.1 | 206 | 0 | 51.4 | 0 | 0 | 0 |
|  | 70.2 | 45.8 | 174 | 0 | 43.5 | 0 | 0 | 0 |
|  | 73.5 | 47.9 | 182 | 0 | 45.5 | 0 | 0 | 0 |
|  | 54 | 35.2 | 134 | 0 | 33.5 | 0 | 0 | 0 |
|  | 91.1 | 59.4 | 226 | 0 | 56.4 | 0 | 0 | 0 |
|  | 75.7 | 49.4 | 188 | 0 | 46.9 | 0 | 0 | 0 |
|  | 72.6 | 47.4 | 180 | 0 | 45.0 | 0 | 0 | 0 |
|  | 63.5 | 41.4 | 157 | 0 | 39.3 | 0 | 0 | 0 |
|  | 60.9 | 39.7 | 151 | 0 | 37.7 | 0 | 0 | 0 |
|  | 58 | 37.8 | 144 | 0 | 35.9 | 0 | 0 | 0 |
|  | 48 | 31.3 | 119 | 0 | 29.7 | 0 | 0 | 0 |
|  | 66.6 | 43.4 | 165 | 0 | 41.3 | 0 | 0 | 0 |
|  | 64.8 | 42.3 | 161 | 0 | 40.2 | 0 | 0 | 0 |
|  | 63.8 | 41.6 | 158 | 0 | 39.5 | 0 | 0 | 0 |
| Mean | 67.2 | 43.8 | 166 | 0 | 41.6 | 0 | 0 | 0 |
| SD | 10.4 | 6.8 | 26 | 0 | 6.4 | 0 | 0 | 0 |
| Min | 48.0 | 31.3 | 119 | 0 | 29.7 | 0 | 0 | 0 |
| Max | 91.1 | 59.4 | 226 | 0 | 56.4 | 0 | 0 | 0 |
