# Supplementary material for: Temporal Change in Biomarkers of Bone Turnover Following Late Evening Ingestion of a Calcium-Fortified, Milk-Based Protein Matrix in Postmenopausal Women with Osteopenia
Source: Nutrients. 2019 Jun 23;11(6):1413. doi: 10.3390/nu11061413 (PMC6627915; doi:10.3390/nu11061413)
Supplement: Supplementary file 1 [file nutrients-11-01413-s001.zip › Supplementary Table 4.docx]

**Supplementary Table S4**: Individual standardised diets for the 2 days of the trial.

|  | **Individual Standardised Diet** | | | | | | | |
| --- | --- | --- | --- | --- | --- | --- | --- | --- |
|  | BM (kg) | Energy (kcal) | Protein (g) | Protein (g/kg) | Carbohydrate (g) | Fat (g) | Calcium (mg) | Vitamin D (ug) |
|  | 69.4 | 1818 | 84.3 | 1.2 | 129.6 | 88.9 | 745 | 11.0 |
|  | 59.5 | 1839 | 79.1 | 1.3 | 166.1 | 80.7 | 1012 | 7.7 |
|  | 83 | 1849 | 79.1 | 1.0 | 167.4 | 81.9 | 1013 | 7.7 |
|  | 70.2 | 1788 | 80.7 | 1.1 | 164.7 | 77.2 | 785 | 10.3 |
|  | 73.5 | 1854 | 91.9 | 1.3 | 196.9 | 104.1 | 973 | 1.9 |
|  | 54 | 1799 | 86.0 | 1.6 | 158.8 | 88.1 | 752 | 11.2 |
|  | 91.1 | 1806 | 90.2 | 1.0 | 195.8 | 101.0 | 731 | 1.7 |
|  | 75.7 | 1877 | 86.0 | 1.1 | 132.0 | 93.2 | 753 | 11.2 |
|  | 72.6 | 1856 | 80.6 | 1.1 | 164.7 | 77.4 | 783 | 10.3 |
|  | 63.5 | 1850 | 95.2 | 1.5 | 196.4 | 103.6 | 518 | 4.8 |
|  | 60.9 | 1803 | 82.7 | 1.4 | 159.2 | 88.5 | 974 | 8.3 |
|  | 58 | 1906 | 82.4 | 1.4 | 166.1 | 80.6 | 792 | 10.6 |
|  | 48 | 1834 | 79.1 | 1.6 | 165.6 | 80.2 | 1013 | 7.7 |
|  | 66.6 | 1873 | 86.0 | 1.3 | 131.6 | 92.8 | 754 | 11.2 |
|  | 64.8 | 1918 | 95.2 | 1.5 | 168.5 | 107.7 | 517 | 4.8 |
|  | 63.8 | 1816 | 84.3 | 1.3 | 129.4 | 88.6 | 746 | 11.0 |
| Mean | 67.2 | 1843 | 85.2 | 1.296 | 162.1 | 89.7 | 804 | 8.2 |
| SD | 10.4 | 37 | 5.2 | 0.19 | 21.8 | 9.7 | 153 | 3.2 |
| Min | 48.0 | 1788 | 79.1 | 1.0 | 129.4 | 77.2 | 517 | 1.7 |
| Max | 91.1 | 1918 | 95.2 | 1.6 | 196.9 | 107.7 | 1013 | 11.2 |
